# Identification of 4-genes model in papillary renal cell tumor microenvironment based on comprehensive analysis

**DOI:** 10.1186/s12885-021-08319-0

**Published:** 2021-05-17

**Authors:** Liang Luo, Haiyi Zhou, Hao Su

**Affiliations:** 1 Department of Urology, The Third Affiliated Hospital, Sun Yat-sen University, Tianhe Road 600, Guangzhou, 510630 China; 2 Department of Gynecology of Traditional Chinese Medicine, Shanxi Academy of Traditional Chinese Medicine, Taiyuan, 030000 China

**Keywords:** Papillary renal cell carcinoma, Tumor microenvironment, Hub genes, Prognosis

## Abstract

**Background:**

The tumor microenvironment acts a pivotal part in the occurrence and development of tumor. However, there are few studies on the microenvironment of papillary renal cell carcinoma (PRCC). Our study aims to explore prognostic genes related to tumor microenvironment in PRCC.

**Methods:**

PRCC expression profiles and clinical data were extracted from The Cancer Gene Atlas (TCGA) and Gene Expression Omnibus (GEO) database. Immune/stromal scores were performed utilizing the ESTIMATE algorithm. Three hundred fifty-seven samples were split into two groups on the basis of median immune/stromal score, and comparison of gene expression was conducted. Intersect genes were obtained by Venn diagrams. Hub genes were selected through protein-protein interaction (PPI) network construction, and relevant functional analysis was conducted by DAVID. We used Kaplan–Meier analysis to identify the correlations between genes and overall survival (OS) and progression-free survival (PFS). Univariate and multivariate cox regression analysis were employed to construct survival model. Cibersort was used to predict the immune cell composition of high and low risk group. Combined nomograms were built to predict PRCC prognosis. Immune properties of PRCC were validated by The Cancer Immunome Atlas (TCIA).

**Results:**

We found immune/stromal score was correlated with T pathological stages and PRCC subtypes. Nine hundred eighty-nine differentially expressed genes (DEGs) and 1169 DEGs were identified respectively on the basis of immune and stromal score. Venn diagrams indicated that 763 co-upregulated genes and 4 co-downregulated genes were identified. Kaplan-Meier analysis revealed that 120 genes were involved in tumor prognosis. Then PPI network analysis identified 22 hub genes, and four of which were significantly related to OS in patients with PRCC confirmed by cox regression analysis. Finally, we constructed a prognostic nomogram which combined with influence factors.

**Conclusions:**

Four tumor microenvironment-related genes (CD79A, CXCL13, IL6 and CCL19) were identified as biomarkers for PRCC prognosis.

**Supplementary Information:**

The online version contains supplementary material available at 10.1186/s12885-021-08319-0.

## Introduction

The incidence of renal cell carcinoma (RCC) is approximately 431,288 in 2020, accounting for 2.2% of common malignant neoplasm on a global scale [1]. Clear cell, papillary, and chromophobe renal cell carcinoma are three main histologic subtypes of RCC. Study reported papillary renal cell carcinoma (PRCC) is the second most common type [2]. Clinicopathological features are the main diagnostic criteria for PRCC, but their predictive outcome is not accurate because of the lack of consistent standards [3]. Surgery is the primary choice of treatment for early stage PRCC, and comprehensive therapeutic approaches which include surgery, and immunotherapy are applied for advanced stage patients [4]. However, the prognosis for PRCC is various on account of tumor metastasis and complications. The World Health Organization (WHO) classification (2016), according to different clinicopathological and immunohistochemical features, split PRCC into two subtypes: type 1 and type 2 [5]. In general, the prognosis of type 2 PRCC was poorer than type 1 [6].

Tumor microenvironment (TME) is an intricate system comprised of tumor cells, surrounding immune and inflammatory cells, tumor-related fibroblasts and nearby stromal tissues, and varieties of cytokines and chemokines [7]. Relevant studies have indicated macrophages play a crucial part in tumor initiation and invasion of adjacent tissues [8]. Tumor-related fibroblasts, a kind of stromal cell, affect cancer progression partly by interacting with tumor cells and immune regulation [9]. Studies have demonstrated the highly enriched CD8+ T cells in PRCC are significantly connected with tumor development, progression, and mortality [10]. Yoshihara et al. put forward the ESTIMATE algorithm that utilizes gene expression patterns to calculate the immune/stromal scores in different tumor tissues [11]. Accumulating evidence showed ESTIMATE helped to clarify the importance of TME in numerous cancers [12–14].

In this study, we extracted PRCC datasets from TCGA and GEO and obtained relevant immune and stromal score calculated by ESTIMATE. After a series of bioinformatics analyses, several tumor microenvironment-related genes in connection with the prognosis of PRCC patients were selected and validated, and were used to establish nomograms as potential tools to evaluate the outcomes of PRCC.

## Materials and methods

### Data preparation

Level_3 gene expression patterns for PRCC was downloaded from TCGA via UCSC Xena (http://xena.ucsc.edu/). Another dataset (GSE2748) was obtained from the GEO database. And corresponding clinical information including overall survival (OS) time and status was extracted from the ONCOMINE database (www.oncomine.org/). Immune and stromal scores were calculated by ESTIMATE packages of R.

### Screening of differentially expressed genes (DEGs)

PRCC samples were split into high and low groups on the basis of median immune/stromal score, limma package of R was utilized to compare DEGs, |Log2FC| > 1 and FDR < 0.05 were deemed significant. Volcano plot was drawn by the ggplot2 package of R.

### Survival analysis

We have adopted Kaplan–Meier analysis to evaluate the relations between DEGs and OS. And Tarone-Ware test was applied for comparing the differences. *P* < 0.05 was considered statistically significant.

### PPI construction and hub genes selection

The String database (https://string-db.org/) was used to analyze the molecular interactions of DEGs and construct a PPI network visualized by Cytoscape (v3.7.2). Then we used the MCODE tool to find densely connected hub genes based on topology.

### Functional enrichment analysis

Gene ontology (GO) analysis was performed by DAVID (http://david.abcc.ncifcrf.gov), comprising three aspects: biological process, cellular component, and molecular function. And Kyoto Encyclopedia of Genes and Genomes (KEGG) analysis was applied for searching functional pathways of hub genes. *P* < 0.05 was deemed statistically significant.

### Abundance analysis of tumor-infltrating immune cells and immunophenoscore (IPS) analysis

CIBERSORT algorithm (http://cibersortx.stanford.edu) was used to estimate the composition of 22 immune cell types of PRCC. The percentages of these types were visualized in a bar graph by using ggplot2 package of R. Immunophenogram of TCIA (https://tcia.at/), influenced by four factors: MHC molecules, immunomodulators, effector cells and suppressor cells, was applied to evaluating the immune properties of PRCC. And IPS scores are positively associated with the immunogenicity.

### Nomogram building

To construct a nomogram to predict patients’ OS and PFS of PRCC, the C index was used to evaluate the discriminating ability, and the calibration chart was drawn to evaluate the accuracy of the nomogram. The rms package of R was used to build the nomogram.

### Statistical analysis

Statistical differences between 2 groups were identified by unpaired Student’s t test or Mann Whitney U test. 1-way ANOVA test and Kruskal-Wallis test were applied when more than 2 groups were compared. Based on accessible clinical data, we used univariate and multivariate cox regression analysis to identify the prognostic values of certain hub genes. Kaplan–Meier analysis was used to estimate the OS and PFS. All statistical analysis was conducted by software packages of R (v3.6.3).

## Results

### Immune/stromal score were significantly associated with T pathological stage and PRCC subtypes

**Fig. 1:**
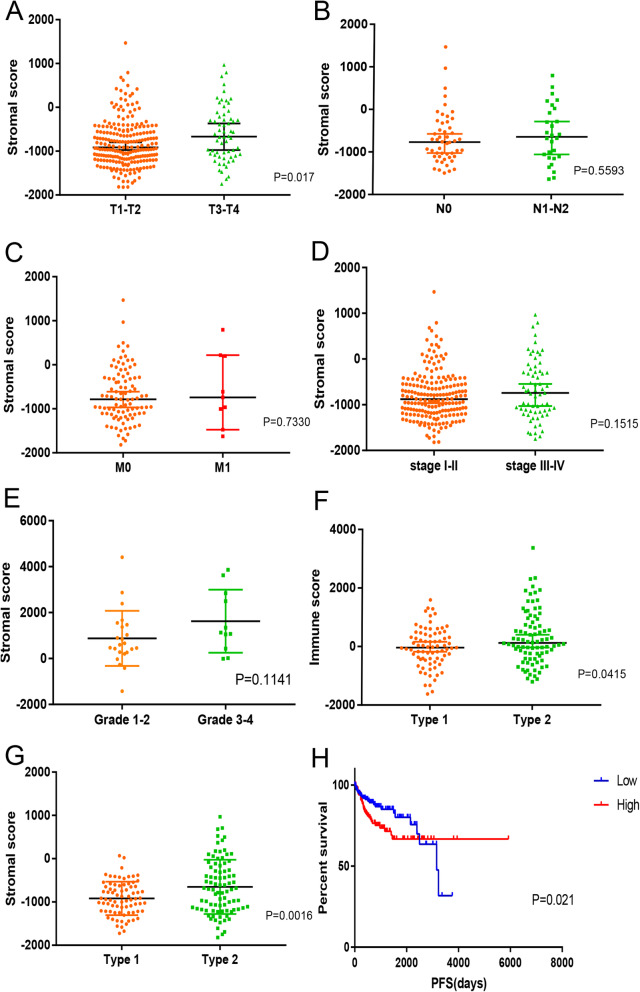
The correlations between the stromal score and clinical characteristics, and OS analysis. **a**–**e**. The relationship between stromal score and T stage, N stage, M stage, clinical stage and grade. **f**, **g**. The relationship between immune/stromal score and PRCC subtypes. **h**. Kaplan-Meier analysis of stromal score with PRCC patients

We downloaded 323 and 34 PRCC samples from the TCGA and GEO database. Gene expression patterns and clinical characteristics were included. Based on the ESTIMATE algorithm, immune score (− 1952.04 to 3372.51) and stromal score (− 1821.04 to 1469.55) of 291 samples of TCGA and 34 samples of GEO were calculated. We attempted to analyze the relations between immune/stromal score and TNM, stage, subtype and grade (Fig. 1a-e). Results showed the differences in the stromal score of T pathological stages were significant (T3–4 > T1–2, *P* < 0.05). And the average immune scores of type 2 were higher than type 1 (*P* < 0.05). Similarly, type 2 had higher stromal scores compared to type 1 (*P* < 0.05, Fig. 1f, g). Finally, we divided the samples into two groups on the basis of median immune/stromal score, and revealed stromal score was significantly associated with PFS (*P* = 0.021, Fig. 1h). However, no significant differences were found in OS via Kaplan–Meier analysis.

### Identification of DEGs with immune/stromal score

To determine whether all genes were associated with immune/stromal score, gene expression profile of 291 patients were analyzed. We mapped volcano plots with the cutoffs of |Log2FC| > 1 and FDR < 0.05 (Fig. S1A, B). Nine hundred eighty-three upregulated and 6 downregulated genes were screened by comparing the high and low immune scores. Meanwhile, we compared 2 groups of cases dichotomized by high and low stromal scores, and extracted 1142 upregulated and 27 downregulated genes. Venn diagrams identified 763 upregulated common genes and 4 downregulated common genes (Fig. S1C, D, Supplementary Table S1). GO analysis showed 763 common upregulated genes were mostly associated with immune and inflammatory response, chemokine activity, IgG binding and cell adhesion molecule binding (Fig. S1 E-G).

### Association of upregulated common genes with OS

In order to obtain the relationships between each gene and OS, Kaplan–Meier was applied for survival analysis of 763 upregulated genes, and the impact of 120 genes (Supplementary Table S2) on OS was statistically significant.

### Selection of molecular complexes from protein interaction networks

To study protein interactions of 120 genes, String tool was performed to structure a PPI network comprised of 84 nodes and 297 edges. Then we used MCODE to select prominent modules: module 1, composed of 17 hub genes (CXCR5, CD19, PDCD1, IL21R, GZMB, TNFRSF9, CD38, CXCL10, SELL, LAG3, CD44, CD80, CCL21, ITGA4, CCL19, IL6, and CXCL13, Fig. S2A) and module 2, including 5 hub genes (BLK, CD79A, FCRLA, MS4A1 and POU2AF, Fig. S2B), which were strongly associated with other genes, suggesting that they might play an important role in PRCC.

### Functional enrichment analysis of valuable genes

To understand whether 22 genes have an impact on the TME, we performed KEGG analysis by DAVID, and the results showed that these genes mainly participated in the following pathways: cytokine-cytokine receptor interaction, chemokine signaling pathway and primary immunodeficiency (Fig. S2C).

### Construction and validation of a survival model to predict prognosis of PRCC patients

**Fig. 2:**
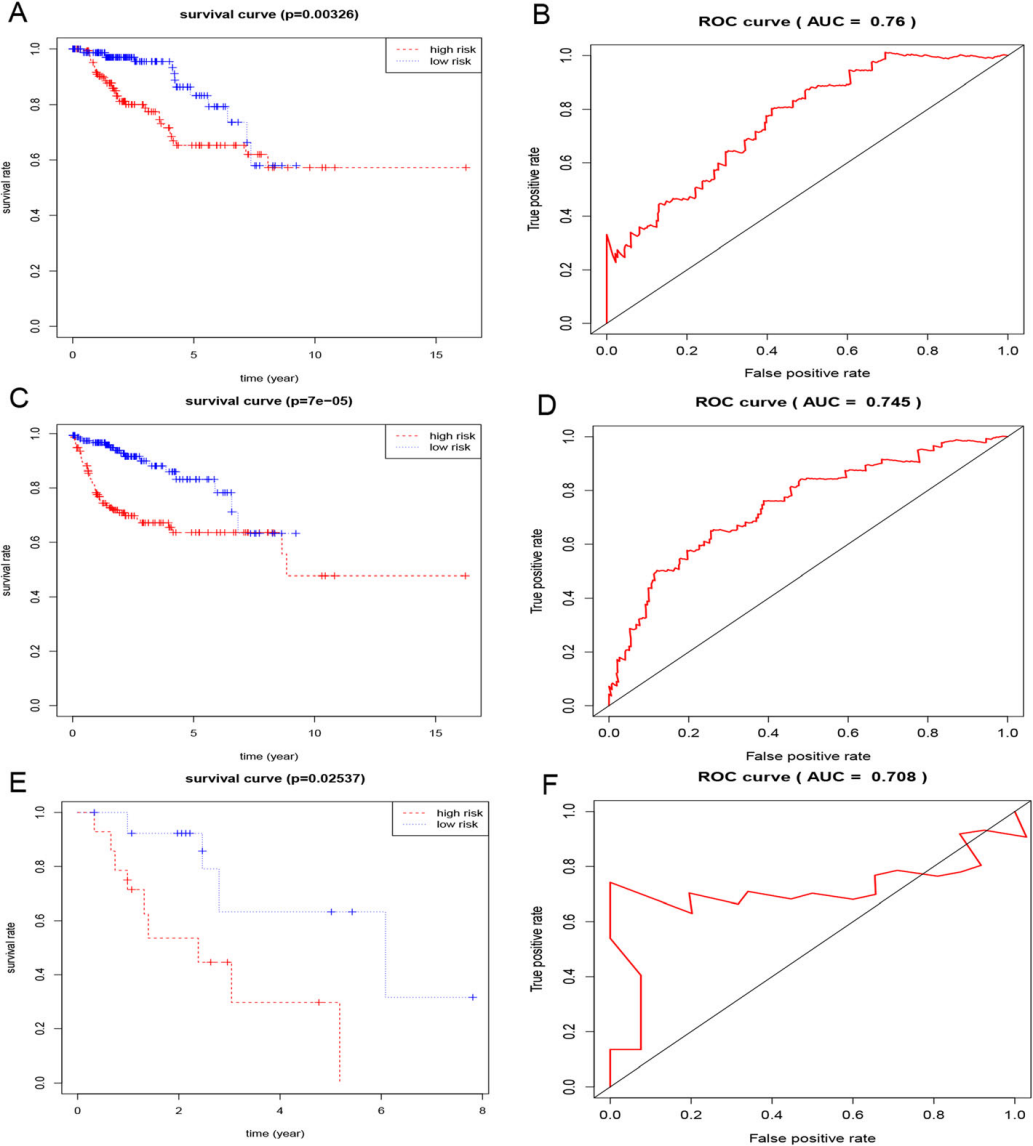
Kaplan-Meier survival and ROC analysis.. OS and PFS were compared respectively between low and high risk group of TCGA samples (**a**, **c**), OS was compared between low and high risk group of GEO samples (**e**), ROC of 4-gene model of OS (**c**) and PFS (**d**) analysis of TCGA samples and OS analysis of GEO samples (**f**)

Three hundred twenty-three PRCC samples from TCGA and 34 samples from GEO were used as train and test group. Univariate cox regression analysis has approved all of 22 genes were significantly with OS (Supplementary Table S3). Then, we applied multivariate cox regression analysis to confirm 4 genes (CD79A, CXCL13, IL6 and CCL19) significantly related with patients’ prognosis (Supplementary Table S3 and S4) and constructed a formula for risk score calculation after extracting the coefficients from the results: expression level of CD79A * (− 0.24020) + CXCL13 * 0.22031+ IL6 * 0.12025+ CCL19 * 0.17997). According to median risk score, 323 PRCC samples from TCGA were divided into high and low risk group, Kaplan–Meier survival curves both indicated patients’ OS and PFS in high risk group were worse than that in low risk group (*p* < 0.05, Fig. 2a, c). And area under curve (AUC) related to OS and PFS was 0.76 and 0.745 (Fig. 2b, d). Similarly, 34 samples from GEO were analyzed, and AUC was 0.708 (Fig. 2e, f). We depicted ROC curves to discuss the predictive ability of this gene model in different subgroups of PRCC, such as subtype, grade, pathologic stage, gender and age. And the results demonstrated the immune signature well performed in age > =65, female and type 1 PRCC (Fig. S3A-D). Compared with clinical characteristics (age, gender, TNM, grade and stage), the 4-genes model showed a great performance in PRCC prognosis (Fig. S3E, F). Then, the relationship between the TMB-signature and clinicopathological factors were analyzed, and the results indicated significant differences were observed in gender, TNM, stage and type (Fig. S4).

### Immune infiltration and IPS analysis

**Fig. 3:**
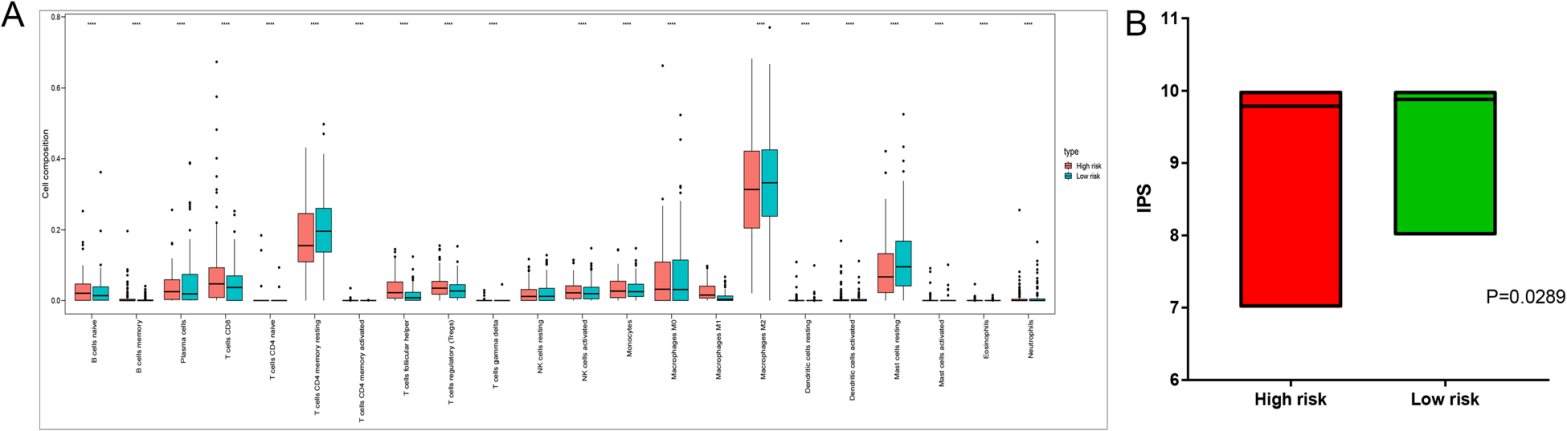
Immune cell composition analysis and the difference analysis of IPS. **a** Box plot of 22 immune cells proportion between low and high risk group of 4-gene model. **b** the difference analysis of IPS between low and high risk group of 4-gene model

In order to predict the composition of immune cells in TCGA samples, we divided patients into low and high risk group based on median risk score. The composition of 22 types of immune cells in 2 groups was depicted in the bar graph (Fig. 3a) which indicated low risk group was connected with recruitment of plasma cell, resting memory CD4+ T cell, macrophage M0/2, and resting mast cell (*P* < 0.05). We used IPS to predict the potential response of ICI (immune checkpoint inhibitors) for PRCC patients and investigated the association between IPS and 4-genes model of PRCC. Statistical analysis indicated that the IPS of low risk group was higher than high risk group (Fig. 3b). Conclusively, low risk group of immune-associated signature might have stronger immunogenicity.

### Construction of predictive nomogram

**Fig. 4:**
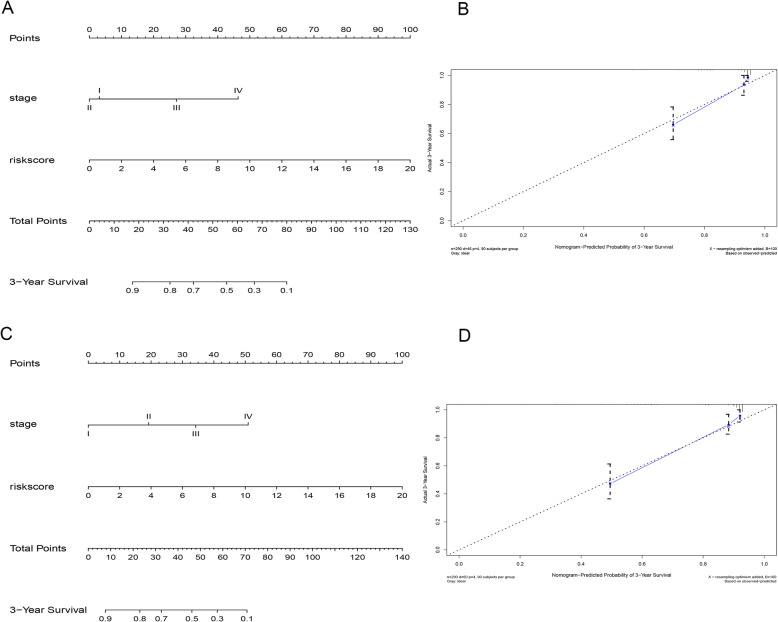
Nomogram for predicting OS **(a**) and PFS **(c)** for PRCC patients of TCGA based on risk score and stage. The calibration plot for internal validation of the nomogram **(b**, **d)**

By comparing the C-index and removing invalid variables, we constructed a nomogram to predict patients’ OS and PFS of PRCC which combined with two independent factors including stage and risk score (Fig. 4). The calibration curve showed the nomogram had the best performance with the C-index of 0.855 and 0.834. Conclusively, this nomogram could be a potential tool to evaluate the outcomes of PRCC.

## Discussion

Recently, TME has been identified to have profound significance and effect on the occurrence, progression, and treatment of tumors, and it is extremely necessary to study its related mechanism. Study has shown changes in mesenchymal stromal cell differentiation promoted the progression of multiple tumors by altering the tumor microenvironment [15]. Maeda-Otsuka et al. demonstrated hypoxia could accelerate the proliferation and migration of angiosarcoma by regulating tumor microenvironment [16]. Bader et al. reported that TME was closely linked to immunotherapy, and the understanding of TME could better guide treatment and achieve precision therapy [17]. Meanwhile, study indicated that immunotherapy was one of the promising options for advanced stage PRCC patients [18]. This study aimed to identify genes in connection with tumor microenvironment and significantly with OS in PRCC patients.

Firstly, we discovered that the differences in the stromal score of T pathological stages were statistically significant, indicating that stromal cells of TME were meaningful in the correlation of T pathological stages. Relevant study has demonstrated that changes in stromal components, such as the presence of myofibroblasts, initiated tumor invasion, and metastasis [19]. Delahunt reported type 1 PRCC inclined to be localized in kidney whereas type 2 generally tended to invade the surrounding organs and had a poor prognosis [20]. Results showed the mean immune/stromal scores of type 2 PRCC cases were significantly higher than type 1, which indicated higher immune/stromal scores were associated with type 2 PRCC, and might be connected with more aggressive biological behaviors. GO analysis of genes co-upregulated in the immune/stromal groups indicated that they were involved in immune and inflammatory responses, suggesting that most of the genes were related to TME. Then, Kaplan–Meier analysis of 763 upregulation genes showed that 120 genes were associated with PRCC prognosis. Combined with PPI network construction and significant modules extraction, we obtained 22 hub genes. Then KEGG enrichment analysis showed that they were active in immune-related pathways, such as cytokine-cytokine receptor interaction, chemokine signaling pathway and primary immunodeficiency. Univariate and multivariate regression analysis put forward a four-genes (CD79A, CXCL13, IL6 and CCL19) survival model based on 323 PRCC samples from the TCGA database, of which AUC was 0.76 of OS and 0.745, and confirmed by a set of GEO data, with the AUC of 0.708. Based on risk score and pathologic stage, we constructed two nomograms separately associated with patients’ OS and PFS. According to IPS program, results showed IPS was significantly higher in low risk group, which indicated lower expression level of 4 immune-related genes were connected with weaker immunogenicity.

Among the 4 genes, Certain studies have demonstrated that CCL19 could act as an immunomodulator by activating dendritic cells, T and B cells in secondary lymphoid tissue to modulate primary (or secondary) adaptive immune responses [21]. Besides, overexpression of CCL19 was discovered to be implicated in tumor progression in cervical cancer, but might be contribute to anti-vascular treatment in colorectal cancer through inhibiting angiogenesis [22, 23]. Numerous studies have shown that CXCL13, as a chemokine secreted by stromal cells was implicated in the occurrence, invasion, and lymph node metastasis of tumors by binding to its receptor CXCR5 [24–27]. Rachana et al. reported it could be a novel and compelling target for prostate cancer therapy [28]. Several studies indicated that CD79A was connected with aggressive hematological malignancy, could be a popular marker in the detection and treatment of hematopathy such as Hodgkin’s lymphoma and B-cell lymphoma [29–31]. IL6, a proinflammatory cytokine, has been reported to be influenced by cancer-associated fibroblasts (CAFs) in TME, and participated in the process of human tumor immunity improvement, tumor metastasis and colonization [32–34].

The interaction of PRCC and its TME affected the whole process from tumor occurrence and progress to metastasis and recurrence, which created plenty of opportunities for diagnosis, treatment and prognosis of PRCC patients. In present study, we attempted to find tumor microenvironment-genes associated with PRCC patients’ survival. Our results may provide some related data to future research on the relations of PRCC and TME. However, the mechanism of PRCC and its microenvironment could be extremely complex, our analysis based on the TCGA and GEO database and bioinformatic tools is only one part of it. Further research and analysis based on large samples will be essential.

## Conclusion

By analyzing the TCGA and GEO database and applying ESTIMATE algorithm and a series of bioinformatic methods, we obtained tumor microenvironment associated genes (CD79A, CXCL13, IL6 and CCL19), which were related to the clinical outcome of PRCC patients. These genes could be used as biomarkers for predicting PRCC prognosis.

## Supplementary Information

**Additional file 1: Fig. S1** Comparison of differentially expressed genes. A. Volcano plots of differentially expressed genes based on immune score. B. Volcano plots of differentially expressed genes based on stromal score. C. Venn plots of co-downregulated genes. D. Venn plots of co-upregulated genes. GO analysis of 763 co-upregulated genes. E. Biological process. F. Cellular component. G. Molecular function.

**Additional file 2: Supplementary Table S1** 763 upregulated common genes and 4 downregulated common genes between TCGA and GEO samples.

**Additional file 3: Supplementary Table S2** 120 genes associated with PRCC overall survival.

**Additional file 4: Fig. S2** PPI networks of significant module. A. module1, B. module2. The node color changes gradually from pink to red indicating the ascending order of the degree of the genes. C KEGG pathway analysis for 17 hub genes.

**Additional file 5: Supplementary Table S3** Univariate cox proportional hazard regression analysis of 22 genes based on TCGA database.

**Additional file 6: Supplementary Table S4** Multivariate cox regression results of 4-gene prognostic model.

**Additional file 7: Fig. S3** ROC curve analysis. The predictive ability of 4-gene model in different ages (A), genders (B), stages (C) and subtypes (D) of PRCC. AUCs of 4-gene model were compared with age, gender, TNM, stage, tumor grade of TCGA (E) and GEO (F).

**Additional file 8: Fig. S4** The association between 4-gene model and clinicopathologic features. A. age, B. gender, C. T, D. M, F. N, G. stage, H. subtype.

## Data Availability

The datasets analyzed during the current study are available in the TCGA and GEO repository. [https://portal.gdc.cancer.gov/, www.ncbi.nlm.nih.gov/geo/query/acc.cgi?acc=GSE2748].

## Abbreviations

PRCC: Papillary renal cell carcinoma
TCGA: The Cancer Gene Atlas
GEO: Gene Expression Omnibus
PPI: Protein-protein interaction
OS: Overall survival
PFS: Progression-free survival
TCIA: The Cancer Immunome Atlas
DEGs: Differentially expressed genes
RCC: Renal cell carcinoma
WHO: World Health Organization
TME: Tumor microenvironment
GO: Gene ontology
KEGG: Kyoto Encyclopedia of Genes and Genomes
IPS: Immunophenoscore,
ICI: Immune checkpoint inhibitors
ROC: Receiver operating characteristic
AUC: Area under curve
CAFs: Cancer-associated fibroblasts
