# Supplementary figures and images for: Identification of 4-genes model in papillary renal cell tumor microenvironment based on comprehensive analysis
Source: BMC Cancer. 2021 May 17;21:553. doi: 10.1186/s12885-021-08319-0 (PMC8127234; doi:10.1186/s12885-021-08319-0)

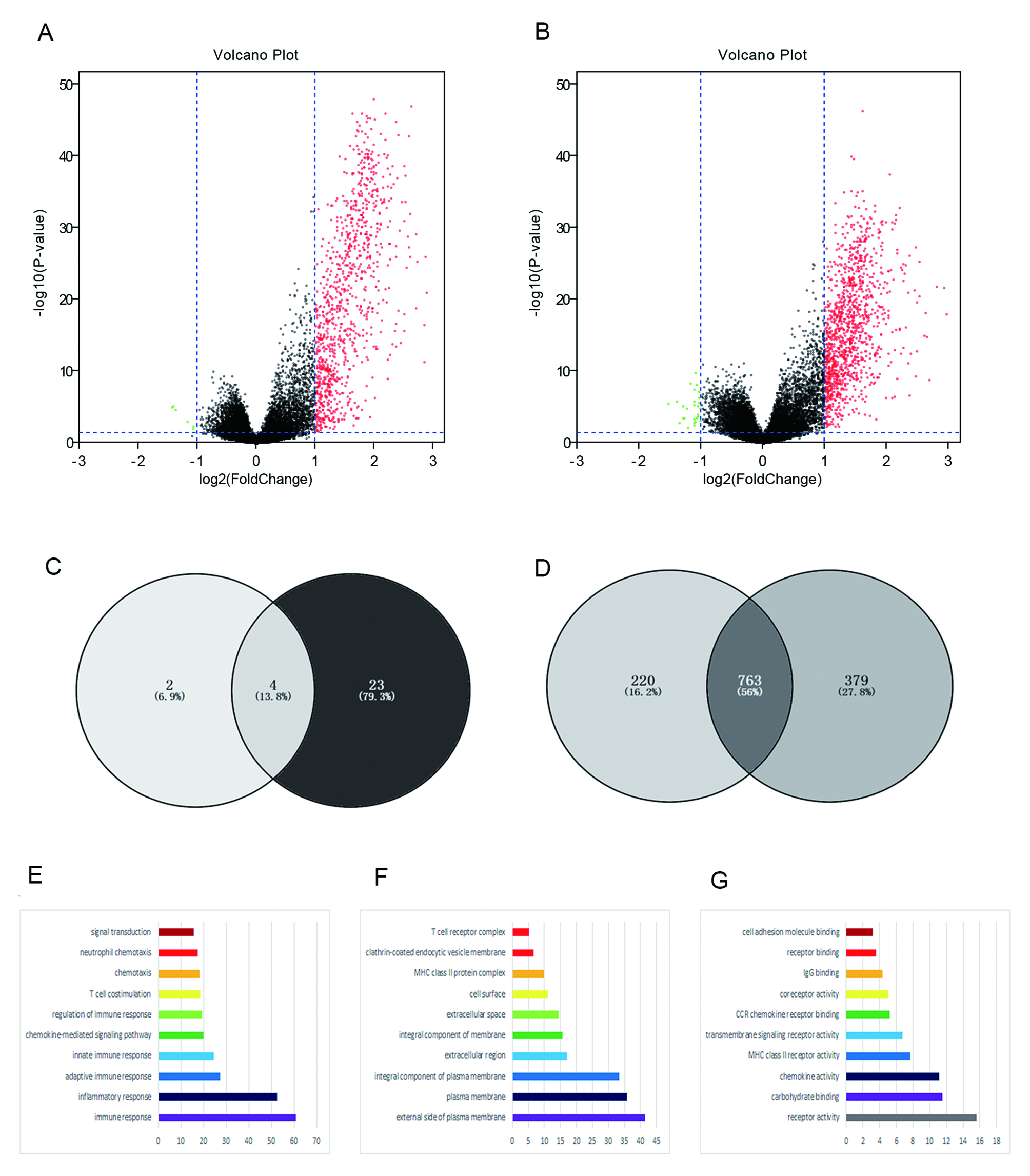

Supplement: Supplementary file 1 — Additional file 1: Fig. S1 Comparison of differentially expressed genes. A. Volcano plots of differentially expressed genes based on immune score. B. Volcano plots of differentially expressed genes based on stromal score. C. Venn plots of co-downregulated genes. D. Venn plots of co-upregulated genes. GO analysis of 763 co-upregulated genes. E. Biological process. F. Cellular component. G. Molecular function. [file 12885_2021_8319_MOESM1_ESM.tif]

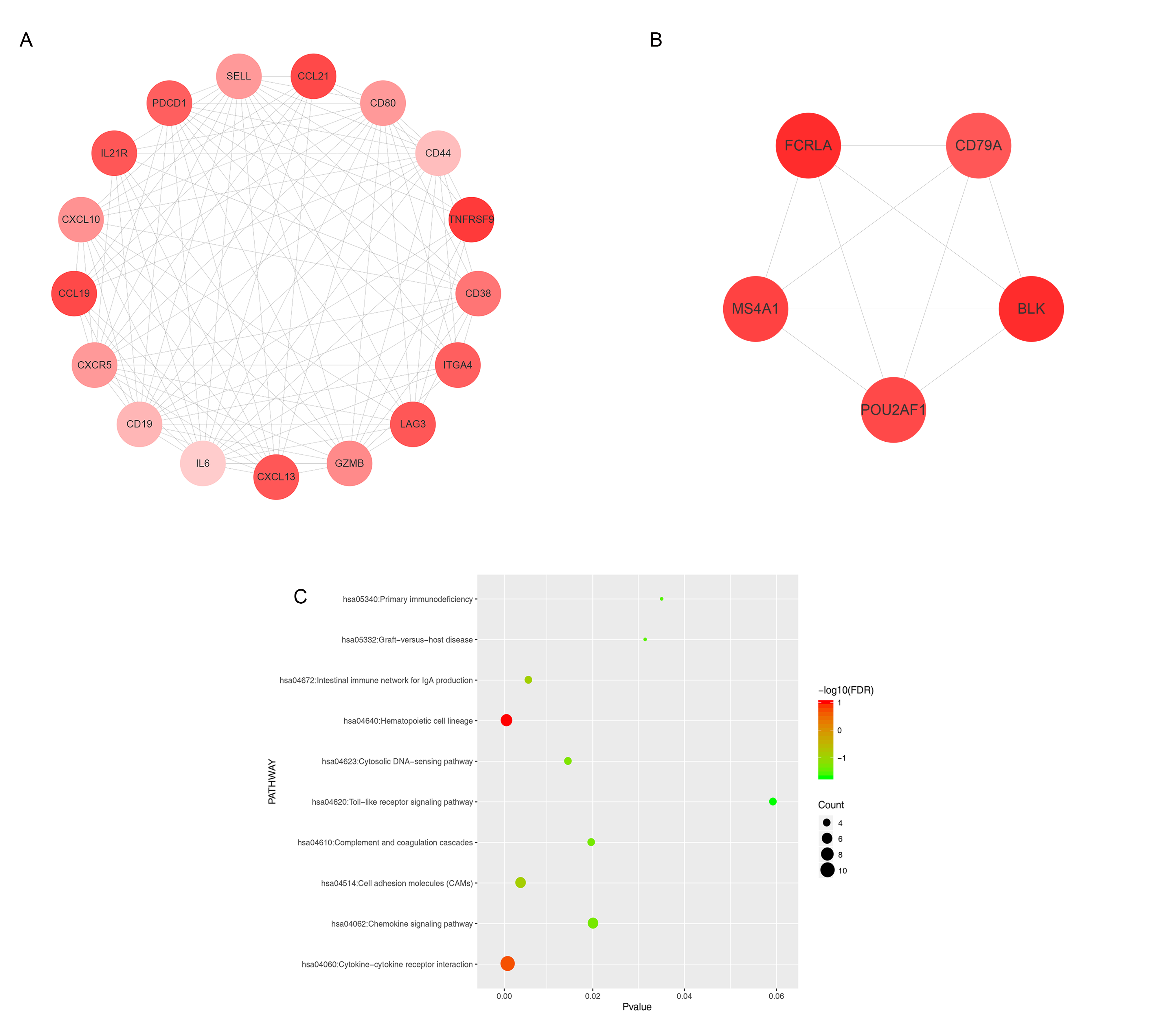

Supplement: Supplementary file 4 — Additional file 4: Fig. S2 PPI networks of significant module. A. module1, B. module2. The node color changes gradually from pink to red indicating the ascending order of the degree of the genes. C KEGG pathway analysis for 17 hub genes. [file 12885_2021_8319_MOESM4_ESM.tif]

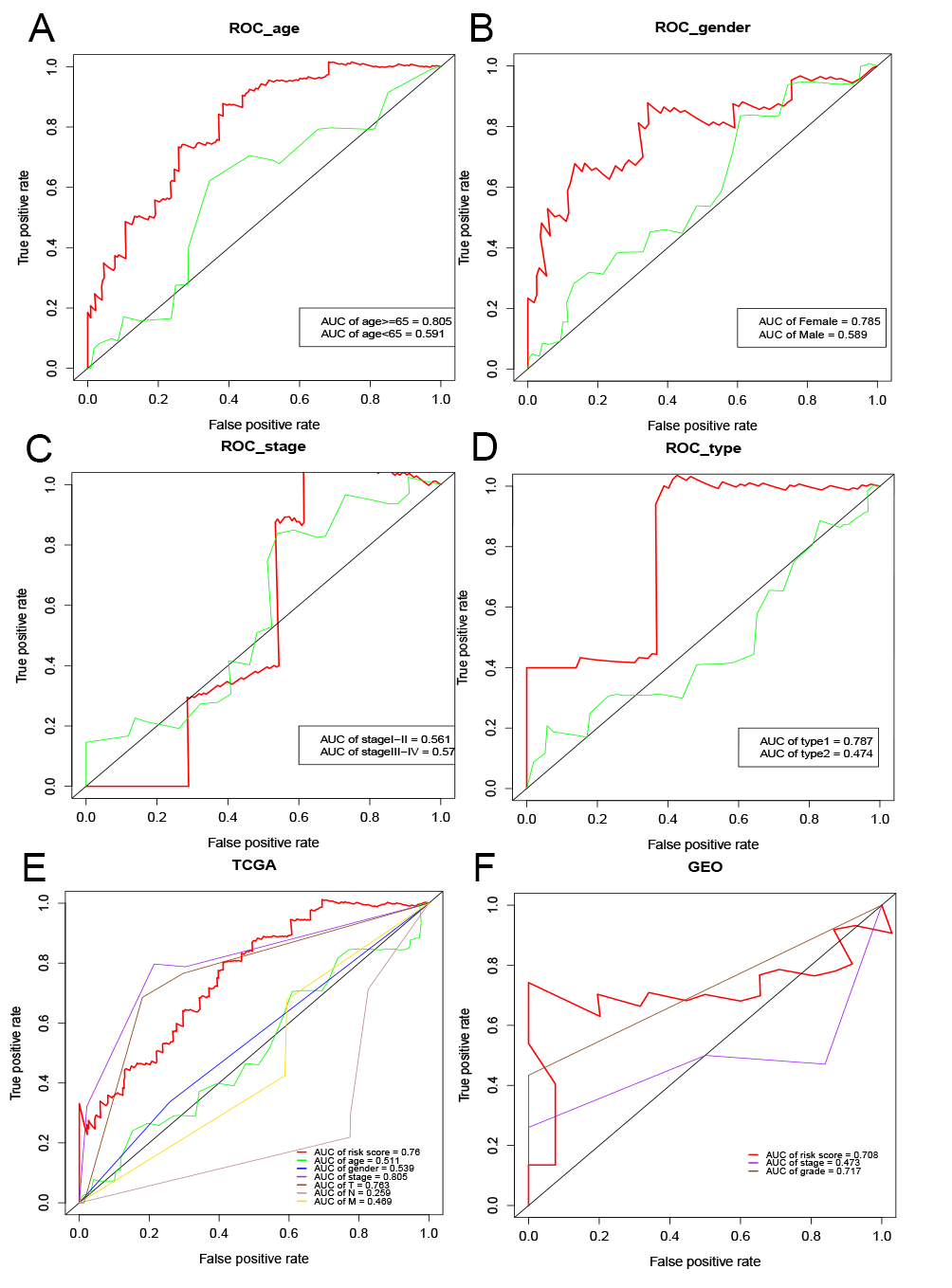

Supplement: Supplementary file 7 — Additional file 7: Fig. S3 ROC curve analysis. The predictive ability of 4-gene model in different ages (A), genders (B), stages (C) and subtypes (D) of PRCC. AUCs of 4-gene model were compared with age, gender, TNM, stage, tumor grade of TCGA (E) and GEO (F). [file 12885_2021_8319_MOESM7_ESM.tif]

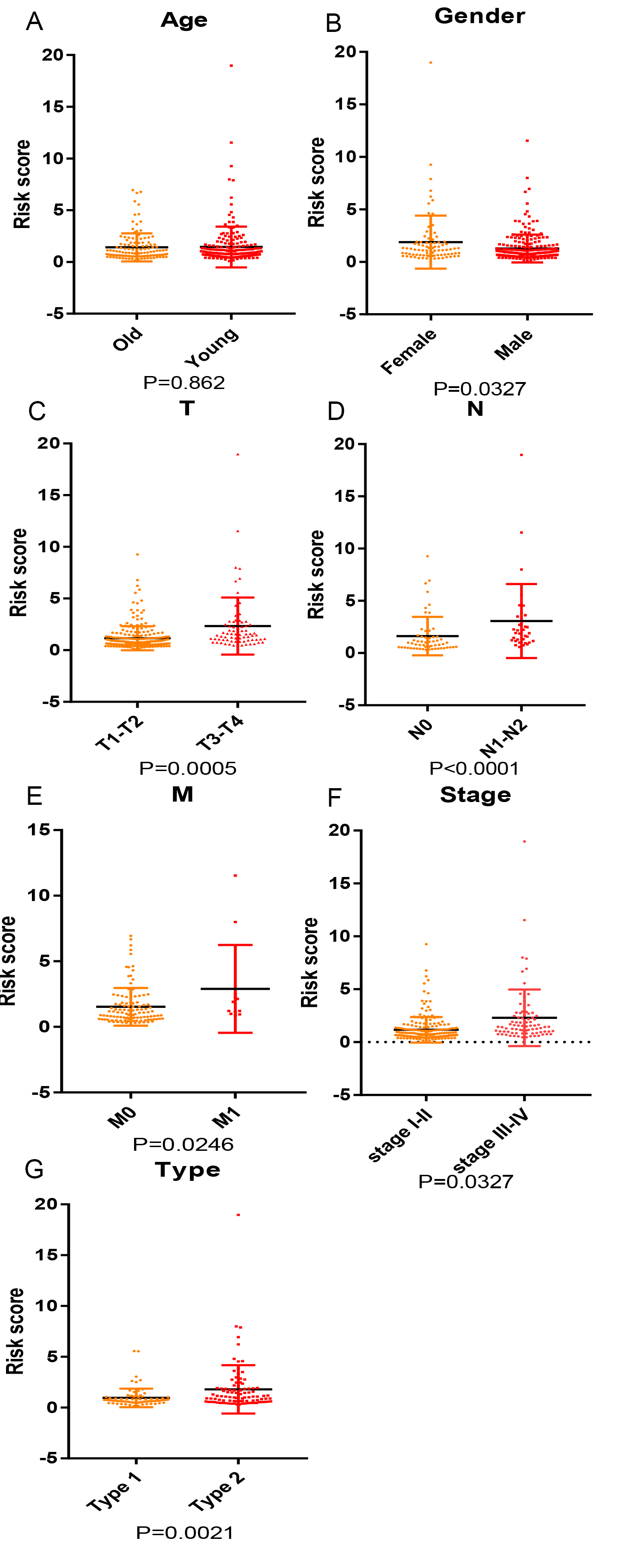

Supplement: Supplementary file 8 — Additional file 8: Fig. S4 The association between 4-gene model and clinicopathologic features. A. age, B. gender, C. T, D. M, F. N, G. stage, H. subtype. [file 12885_2021_8319_MOESM8_ESM.tif]
