# Supplementary material for: Identification of 4-genes model in papillary renal cell tumor microenvironment based on comprehensive analysis
Source: BMC Cancer. 2021 May 17;21:553. doi: 10.1186/s12885-021-08319-0 (PMC8127234; doi:10.1186/s12885-021-08319-0)
Supplement: Supplementary file 2 — Additional file 2: Supplementary Table S1 763 upregulated common genes and 4 downregulated common genes between TCGA and GEO samples. [file 12885_2021_8319_MOESM2_ESM.docx]

Title: Identification of 4-genes model in papillary renal cell tumor microenvironment based on comprehensive analysis

Liang Luo^1^*, Haiyi Zhou^2^, Hao Su^1^

1 Department of Urology, The Third Affiliated Hospital, Sun Yat-sen University, Guangzhou, 510630, China

2 Department of Gynecology of traditional Chinese Medicine, Shanxi Academy of Traditional Chinese Medicine, Taiyuan 030000, China

Corresponding author:

Liang Luo, Department of Urology, The Third Affiliated Hospital, Sun Yat-sen University, Tianhe Road 600, Guangzhou, 510630, China

Telephone: +86-20-85252990; Fax: +86-20-85252678

E-mail: luoliang6@mail2.sysu.edu.cn

**Supplementary Table S1**

**763 upregulated common genes and 4 downregulated common genes between TCGA and GEO samples**

| CREB3L1 | Up-Regulated |
| --- | --- |
| CASS4 | Up-Regulated |
| MMP2 | Up-Regulated |
| CD44 | Up-Regulated |
| ZAP70 | Up-Regulated |
| GRAP2 | Up-Regulated |
| PSTPIP1 | Up-Regulated |
| SPIB | Up-Regulated |
| IL12RB1 | Up-Regulated |
| CXCR4 | Up-Regulated |
| APOB48R | Up-Regulated |
| NCF4 | Up-Regulated |
| CRTAM | Up-Regulated |
| GSTA1 | Up-Regulated |
| IGF1 | Up-Regulated |
| MATK | Up-Regulated |
| CHRDL1 | Up-Regulated |
| NCF1B | Up-Regulated |
| NCF1C | Up-Regulated |
| LILRA4 | Up-Regulated |
| C1R | Up-Regulated |
| SIDT1 | Up-Regulated |
| CD300C | Up-Regulated |
| AMICA1 | Up-Regulated |
| COL22A1 | Up-Regulated |
| HS3ST3A1 | Up-Regulated |
| RTN1 | Up-Regulated |
| RAB42 | Up-Regulated |
| LILRB2 | Up-Regulated |
| SIRPB2 | Up-Regulated |
| UTY | Up-Regulated |
| C1QC | Up-Regulated |
| GZMM | Up-Regulated |
| FCRL6 | Up-Regulated |
| SLC2A5 | Up-Regulated |
| FAM113B | Up-Regulated |
| KBTBD8 | Up-Regulated |
| PLCB2 | Up-Regulated |
| SLC38A5 | Up-Regulated |
| NLRC4 | Up-Regulated |
| FGR | Up-Regulated |
| KCNE1 | Up-Regulated |
| C16orf54 | Up-Regulated |
| AQP9 | Up-Regulated |
| CD4 | Up-Regulated |
| HEPH | Up-Regulated |
| ITLN1 | Up-Regulated |
| DOCK2 | Up-Regulated |
| FAM20A | Up-Regulated |
| HLA-DRB5 | Up-Regulated |
| SPN | Up-Regulated |
| FPR3 | Up-Regulated |
| PRAM1 | Up-Regulated |
| SH2D1A | Up-Regulated |
| CXCL13 | Up-Regulated |
| GIMAP8 | Up-Regulated |
| PLEK | Up-Regulated |
| NLRP3 | Up-Regulated |
| RNASE6 | Up-Regulated |
| SERPINF1 | Up-Regulated |
| CSF3R | Up-Regulated |
| CCL22 | Up-Regulated |
| GPNMB | Up-Regulated |
| CD33 | Up-Regulated |
| PTPN22 | Up-Regulated |
| NAPSB | Up-Regulated |
| RUNX3 | Up-Regulated |
| BASP1 | Up-Regulated |
| FCGR2B | Up-Regulated |
| PRKCB | Up-Regulated |
| GVIN1 | Up-Regulated |
| HCST | Up-Regulated |
| C5AR1 | Up-Regulated |
| SIGLEC8 | Up-Regulated |
| A2M | Up-Regulated |
| PIK3R5 | Up-Regulated |
| ARHGAP30 | Up-Regulated |
| SLCO2B1 | Up-Regulated |
| SIGLEC9 | Up-Regulated |
| ARL11 | Up-Regulated |
| CPNE5 | Up-Regulated |
| CCL4L2 | Up-Regulated |
| AP1S2 | Up-Regulated |
| ADAMDEC1 | Up-Regulated |
| PTGDR | Up-Regulated |
| CST7 | Up-Regulated |
| SELL | Up-Regulated |
| CCL19 | Up-Regulated |
| RFTN1 | Up-Regulated |
| GPR55 | Up-Regulated |
| CLEC4E | Up-Regulated |
| LRRC33 | Up-Regulated |
| TMEM150B | Up-Regulated |
| HTR7 | Up-Regulated |
| PLEKHO1 | Up-Regulated |
| CD38 | Up-Regulated |
| TNFRSF9 | Up-Regulated |
| IKZF1 | Up-Regulated |
| PODN | Up-Regulated |
| LAIR1 | Up-Regulated |
| LOC96610 | Up-Regulated |
| EVI2A | Up-Regulated |
| ROR2 | Up-Regulated |
| GIMAP1 | Up-Regulated |
| GSTA2 | Up-Regulated |
| ALK | Up-Regulated |
| APOE | Up-Regulated |
| ST8SIA4 | Up-Regulated |
| C3 | Up-Regulated |
| LILRB4 | Up-Regulated |
| CES4 | Up-Regulated |
| POU2AF1 | Up-Regulated |
| GSDMA | Up-Regulated |
| FGG | Up-Regulated |
| FABP5 | Up-Regulated |
| CLEC2B | Up-Regulated |
| PLIN2 | Up-Regulated |
| TSPAN8 | Up-Regulated |
| SLC2A3 | Up-Regulated |
| TMEM156 | Up-Regulated |
| C3AR1 | Up-Regulated |
| SAMSN1 | Up-Regulated |
| CD36 | Up-Regulated |
| EMILIN1 | Up-Regulated |
| NAALADL1 | Up-Regulated |
| FCN1 | Up-Regulated |
| DUSP2 | Up-Regulated |
| BIRC7 | Up-Regulated |
| NCF2 | Up-Regulated |
| ADAM19 | Up-Regulated |
| CD6 | Up-Regulated |
| CRHBP | Up-Regulated |
| HLA-DRA | Up-Regulated |
| CTSW | Up-Regulated |
| APOL4 | Up-Regulated |
| SLC9A9 | Up-Regulated |
| RCSD1 | Up-Regulated |
| CXCR2 | Up-Regulated |
| FYB | Up-Regulated |
| RNASE2 | Up-Regulated |
| RHOH | Up-Regulated |
| HVCN1 | Up-Regulated |
| LY86 | Up-Regulated |
| GMFG | Up-Regulated |
| SLAMF1 | Up-Regulated |
| RASGRP2 | Up-Regulated |
| CFH | Up-Regulated |
| NRP2 | Up-Regulated |
| RASSF5 | Up-Regulated |
| FCRL3 | Up-Regulated |
| C13orf18 | Up-Regulated |
| FGB | Up-Regulated |
| IKZF3 | Up-Regulated |
| LAG3 | Up-Regulated |
| FERMT3 | Up-Regulated |
| ITGB1BP3 | Up-Regulated |
| DAPP1 | Up-Regulated |
| PILRA | Up-Regulated |
| MFAP5 | Up-Regulated |
| KIAA0748 | Up-Regulated |
| GPR171 | Up-Regulated |
| C17orf60 | Up-Regulated |
| TM6SF1 | Up-Regulated |
| CHI3L1 | Up-Regulated |
| SIGLEC14 | Up-Regulated |
| CD300A | Up-Regulated |
| CD53 | Up-Regulated |
| CIITA | Up-Regulated |
| CXCL11 | Up-Regulated |
| PNOC | Up-Regulated |
| C6orf105 | Up-Regulated |
| ARHGAP15 | Up-Regulated |
| HLA-DPB2 | Up-Regulated |
| GNB4 | Up-Regulated |
| FCGR1B | Up-Regulated |
| HLA-DQB1 | Up-Regulated |
| LILRA6 | Up-Regulated |
| C12orf59 | Up-Regulated |
| GZMK | Up-Regulated |
| LRRC25 | Up-Regulated |
| TIAM1 | Up-Regulated |
| MNDA | Up-Regulated |
| GFI1 | Up-Regulated |
| HK3 | Up-Regulated |
| EMR1 | Up-Regulated |
| TRAT1 | Up-Regulated |
| MS4A6A | Up-Regulated |
| FAIM3 | Up-Regulated |
| SH2D2A | Up-Regulated |
| ABCD2 | Up-Regulated |
| NKG7 | Up-Regulated |
| HP | Up-Regulated |
| BTK | Up-Regulated |
| CD180 | Up-Regulated |
| TAGAP | Up-Regulated |
| HCK | Up-Regulated |
| MAP4K1 | Up-Regulated |
| CSTA | Up-Regulated |
| NIPAL4 | Up-Regulated |
| TBX21 | Up-Regulated |
| SLC43A3 | Up-Regulated |
| OTOA | Up-Regulated |
| LEF1 | Up-Regulated |
| STEAP4 | Up-Regulated |
| KLHL6 | Up-Regulated |
| TRPV2 | Up-Regulated |
| GTSF1 | Up-Regulated |
| CARD9 | Up-Regulated |
| CTSS | Up-Regulated |
| LILRA2 | Up-Regulated |
| SVEP1 | Up-Regulated |
| MFSD2A | Up-Regulated |
| LCK | Up-Regulated |
| TIFAB | Up-Regulated |
| TIGIT | Up-Regulated |
| KLRK1 | Up-Regulated |
| RAMP1 | Up-Regulated |
| LRRC2 | Up-Regulated |
| GLDN | Up-Regulated |
| SLA | Up-Regulated |
| NCR3 | Up-Regulated |
| ST8SIA1 | Up-Regulated |
| HLA-DPA1 | Up-Regulated |
| CCR2 | Up-Regulated |
| CNRIP1 | Up-Regulated |
| PTCRA | Up-Regulated |
| CXCR2P1 | Up-Regulated |
| TMC8 | Up-Regulated |
| FCER1A | Up-Regulated |
| ANKRD22 | Up-Regulated |
| SELP | Up-Regulated |
| REG1B | Up-Regulated |
| LTB | Up-Regulated |
| TLR8 | Up-Regulated |
| KCNMB1 | Up-Regulated |
| HLA-DOA | Up-Regulated |
| HLA-DMB | Up-Regulated |
| IL8 | Up-Regulated |
| P2RY8 | Up-Regulated |
| ASCL2 | Up-Regulated |
| TNNI2 | Up-Regulated |
| CD7 | Up-Regulated |
| CD27 | Up-Regulated |
| MAN1C1 | Up-Regulated |
| CD207 | Up-Regulated |
| TNFRSF17 | Up-Regulated |
| SIRPG | Up-Regulated |
| S1PR4 | Up-Regulated |
| CD226 | Up-Regulated |
| KYNU | Up-Regulated |
| CD8A | Up-Regulated |
| SLAMF8 | Up-Regulated |
| GPR141 | Up-Regulated |
| IL34 | Up-Regulated |
| KRT36 | Up-Regulated |
| ARHGAP25 | Up-Regulated |
| STX11 | Up-Regulated |
| AMPD1 | Up-Regulated |
| TBXAS1 | Up-Regulated |
| GPR174 | Up-Regulated |
| EMR2 | Up-Regulated |
| PTGS2 | Up-Regulated |
| SLC29A3 | Up-Regulated |
| OSM | Up-Regulated |
| OLR1 | Up-Regulated |
| FCRL2 | Up-Regulated |
| FAM49A | Up-Regulated |
| TRIM63 | Up-Regulated |
| RASAL3 | Up-Regulated |
| NCF1 | Up-Regulated |
| IRAK3 | Up-Regulated |
| CD2 | Up-Regulated |
| GNA15 | Up-Regulated |
| IL21R | Up-Regulated |
| FAM78A | Up-Regulated |
| FCGR3A | Up-Regulated |
| MCHR1 | Up-Regulated |
| CD83 | Up-Regulated |
| PYHIN1 | Up-Regulated |
| NINJ2 | Up-Regulated |
| CTSK | Up-Regulated |
| WDFY4 | Up-Regulated |
| SIGLEC1 | Up-Regulated |
| CCL4 | Up-Regulated |
| MSR1 | Up-Regulated |
| TRPM2 | Up-Regulated |
| WAS | Up-Regulated |
| S100B | Up-Regulated |
| SEMA7A | Up-Regulated |
| TM7SF4 | Up-Regulated |
| MGP | Up-Regulated |
| TAC1 | Up-Regulated |
| LGALS12 | Up-Regulated |
| LAT2 | Up-Regulated |
| PRF1 | Up-Regulated |
| LST1 | Up-Regulated |
| VSIG4 | Up-Regulated |
| ITIH3 | Up-Regulated |
| CMKLR1 | Up-Regulated |
| ABCB5 | Up-Regulated |
| FLI1 | Up-Regulated |
| CD79B | Up-Regulated |
| CAMP | Up-Regulated |
| IL15RA | Up-Regulated |
| SNCA | Up-Regulated |
| ITK | Up-Regulated |
| SRGN | Up-Regulated |
| EPSTI1 | Up-Regulated |
| CAMK1G | Up-Regulated |
| GNG2 | Up-Regulated |
| ACSL5 | Up-Regulated |
| ITGB2 | Up-Regulated |
| FOLR2 | Up-Regulated |
| KIAA1274 | Up-Regulated |
| SIT1 | Up-Regulated |
| DEF6 | Up-Regulated |
| CD3D | Up-Regulated |
| GIMAP6 | Up-Regulated |
| UBD | Up-Regulated |
| EOMES | Up-Regulated |
| GPAT2 | Up-Regulated |
| SNX20 | Up-Regulated |
| C17orf87 | Up-Regulated |
| MFI2 | Up-Regulated |
| TNFRSF8 | Up-Regulated |
| PTGIR | Up-Regulated |
| ART4 | Up-Regulated |
| REG3G | Up-Regulated |
| GPR18 | Up-Regulated |
| NELL2 | Up-Regulated |
| C7 | Up-Regulated |
| S100A8 | Up-Regulated |
| CHIT1 | Up-Regulated |
| ZBP1 | Up-Regulated |
| MRC1 | Up-Regulated |
| AIF1 | Up-Regulated |
| CD86 | Up-Regulated |
| RGS18 | Up-Regulated |
| POU2F2 | Up-Regulated |
| BLK | Up-Regulated |
| GBP4 | Up-Regulated |
| CCL2 | Up-Regulated |
| LILRA5 | Up-Regulated |
| DARC | Up-Regulated |
| NNMT | Up-Regulated |
| SLC7A8 | Up-Regulated |
| AGAP2 | Up-Regulated |
| HLA-DQA1 | Up-Regulated |
| FAM26F | Up-Regulated |
| ABI3 | Up-Regulated |
| NFAM1 | Up-Regulated |
| IL6 | Up-Regulated |
| PTPN7 | Up-Regulated |
| COLEC12 | Up-Regulated |
| BATF | Up-Regulated |
| CARD16 | Up-Regulated |
| SLAMF6 | Up-Regulated |
| IGJ | Up-Regulated |
| PTGER4 | Up-Regulated |
| CCR7 | Up-Regulated |
| XCL2 | Up-Regulated |
| BCL2A1 | Up-Regulated |
| RGS1 | Up-Regulated |
| CR1 | Up-Regulated |
| PRDM1 | Up-Regulated |
| GAPT | Up-Regulated |
| DHRS9 | Up-Regulated |
| FGD2 | Up-Regulated |
| MX2 | Up-Regulated |
| UCP2 | Up-Regulated |
| EFEMP1 | Up-Regulated |
| UBASH3A | Up-Regulated |
| HLA-DRB6 | Up-Regulated |
| ZNF831 | Up-Regulated |
| CYTH4 | Up-Regulated |
| CCRL2 | Up-Regulated |
| FPR1 | Up-Regulated |
| ACAP1 | Up-Regulated |
| CCR4 | Up-Regulated |
| PTPRC | Up-Regulated |
| CXCR5 | Up-Regulated |
| CD14 | Up-Regulated |
| ITGA4 | Up-Regulated |
| ARHGDIB | Up-Regulated |
| XCL1 | Up-Regulated |
| IRF4 | Up-Regulated |
| ITGAM | Up-Regulated |
| SDS | Up-Regulated |
| PDPN | Up-Regulated |
| PARVG | Up-Regulated |
| GPR82 | Up-Regulated |
| P2RY13 | Up-Regulated |
| FASLG | Up-Regulated |
| CD163 | Up-Regulated |
| CD37 | Up-Regulated |
| GIMAP2 | Up-Regulated |
| PRDM8 | Up-Regulated |
| CCL23 | Up-Regulated |
| LOC100233209 | Up-Regulated |
| LUM | Up-Regulated |
| C1S | Up-Regulated |
| PARP15 | Up-Regulated |
| HMOX1 | Up-Regulated |
| FCGR2A | Up-Regulated |
| BMF | Up-Regulated |
| GRAMD1B | Up-Regulated |
| CGNL1 | Up-Regulated |
| CECR1 | Up-Regulated |
| GZMH | Up-Regulated |
| TYROBP | Up-Regulated |
| GPR65 | Up-Regulated |
| OSCAR | Up-Regulated |
| PECAM1 | Up-Regulated |
| MS4A1 | Up-Regulated |
| ADAM6 | Up-Regulated |
| EMB | Up-Regulated |
| FAM101A | Up-Regulated |
| CCL18 | Up-Regulated |
| CD244 | Up-Regulated |
| TPRG1 | Up-Regulated |
| F13A1 | Up-Regulated |
| LSP1 | Up-Regulated |
| ALOX15B | Up-Regulated |
| IL2RA | Up-Regulated |
| FAP | Up-Regulated |
| ARHGAP9 | Up-Regulated |
| CCR1 | Up-Regulated |
| HHEX | Up-Regulated |
| HTR2B | Up-Regulated |
| PTGDS | Up-Regulated |
| BST2 | Up-Regulated |
| FCGR1C | Up-Regulated |
| S100A4 | Up-Regulated |
| IL10 | Up-Regulated |
| CYP2S1 | Up-Regulated |
| ACP5 | Up-Regulated |
| GPR183 | Up-Regulated |
| LILRB1 | Up-Regulated |
| TLR10 | Up-Regulated |
| ISLR | Up-Regulated |
| CD80 | Up-Regulated |
| CHRNA1 | Up-Regulated |
| ICAM4 | Up-Regulated |
| GPR77 | Up-Regulated |
| ALOX5AP | Up-Regulated |
| POSTN | Up-Regulated |
| RIPK3 | Up-Regulated |
| LOC654433 | Up-Regulated |
| FLJ16779 | Up-Regulated |
| ITGAD | Up-Regulated |
| C10orf128 | Up-Regulated |
| CADM3 | Up-Regulated |
| LILRB3 | Up-Regulated |
| SPI1 | Up-Regulated |
| GZMA | Up-Regulated |
| AG2 | Up-Regulated |
| TREM2 | Up-Regulated |
| MS4A4A | Up-Regulated |
| FMO3 | Up-Regulated |
| PIK3CG | Up-Regulated |
| MGC29506 | Up-Regulated |
| CD5L | Up-Regulated |
| NKAIN4 | Up-Regulated |
| LY75 | Up-Regulated |
| CYGB | Up-Regulated |
| LOC653786 | Up-Regulated |
| CPXM1 | Up-Regulated |
| CLECL1 | Up-Regulated |
| ZC3H12D | Up-Regulated |
| TCL1A | Up-Regulated |
| KCNAB2 | Up-Regulated |
| CD79A | Up-Regulated |
| GIMAP7 | Up-Regulated |
| CXCR3 | Up-Regulated |
| CYSLTR1 | Up-Regulated |
| FXYD5 | Up-Regulated |
| CHI3L2 | Up-Regulated |
| CTLA4 | Up-Regulated |
| CBR3 | Up-Regulated |
| PLA2G2D | Up-Regulated |
| VNN1 | Up-Regulated |
| TLR7 | Up-Regulated |
| FGA | Up-Regulated |
| STAB1 | Up-Regulated |
| PLD4 | Up-Regulated |
| DOK3 | Up-Regulated |
| CEACAM21 | Up-Regulated |
| GIMAP4 | Up-Regulated |
| CLEC7A | Up-Regulated |
| FOS | Up-Regulated |
| SLC37A2 | Up-Regulated |
| CCL8 | Up-Regulated |
| TNFSF13B | Up-Regulated |
| CD28 | Up-Regulated |
| SELPLG | Up-Regulated |
| CCDC80 | Up-Regulated |
| DOCK10 | Up-Regulated |
| TBC1D10C | Up-Regulated |
| FAIM2 | Up-Regulated |
| CCL3L1 | Up-Regulated |
| HLA-DPB1 | Up-Regulated |
| GPR34 | Up-Regulated |
| CXCL12 | Up-Regulated |
| SLC43A1 | Up-Regulated |
| CTSE | Up-Regulated |
| LPAR5 | Up-Regulated |
| SASH3 | Up-Regulated |
| OAS2 | Up-Regulated |
| TMEM173 | Up-Regulated |
| IFI30 | Up-Regulated |
| FCGR2C | Up-Regulated |
| BIN2 | Up-Regulated |
| CD74 | Up-Regulated |
| WNT10A | Up-Regulated |
| CORO1A | Up-Regulated |
| SP140 | Up-Regulated |
| CD163L1 | Up-Regulated |
| EVI2B | Up-Regulated |
| TNFSF8 | Up-Regulated |
| RASSF2 | Up-Regulated |
| DNAJC5B | Up-Regulated |
| CRYBB1 | Up-Regulated |
| GPSM3 | Up-Regulated |
| CD52 | Up-Regulated |
| NCKAP1L | Up-Regulated |
| HLA-DQA2 | Up-Regulated |
| CXCL9 | Up-Regulated |
| PID1 | Up-Regulated |
| ADORA3 | Up-Regulated |
| CD19 | Up-Regulated |
| TNFRSF1B | Up-Regulated |
| LGALS9 | Up-Regulated |
| GZMB | Up-Regulated |
| FCGR3B | Up-Regulated |
| LILRA1 | Up-Regulated |
| PRR5L | Up-Regulated |
| CD300LB | Up-Regulated |
| KAL1 | Up-Regulated |
| ELMO1 | Up-Regulated |
| CYTIP | Up-Regulated |
| CYBB | Up-Regulated |
| LTF | Up-Regulated |
| SLC24A4 | Up-Regulated |
| GGTA1 | Up-Regulated |
| C2 | Up-Regulated |
| GAL3ST4 | Up-Regulated |
| PLA2G7 | Up-Regulated |
| CD247 | Up-Regulated |
| LGALS9C | Up-Regulated |
| CXorf21 | Up-Regulated |
| C1QB | Up-Regulated |
| LRMP | Up-Regulated |
| LYL1 | Up-Regulated |
| CAMK4 | Up-Regulated |
| SIGLEC10 | Up-Regulated |
| P2RY10 | Up-Regulated |
| CETP | Up-Regulated |
| APOC2 | Up-Regulated |
| LOXL3 | Up-Regulated |
| INMT | Up-Regulated |
| P2RX1 | Up-Regulated |
| SLC15A3 | Up-Regulated |
| CD48 | Up-Regulated |
| CELF2 | Up-Regulated |
| SAA1 | Up-Regulated |
| REG1A | Up-Regulated |
| ITM2A | Up-Regulated |
| ITGAX | Up-Regulated |
| CEBPA | Up-Regulated |
| SLAMF7 | Up-Regulated |
| PDE4B | Up-Regulated |
| GPR84 | Up-Regulated |
| FCRL5 | Up-Regulated |
| 1-Mar | Up-Regulated |
| IL1RN | Up-Regulated |
| C5orf20 | Up-Regulated |
| C1orf59 | Up-Regulated |
| MAFB | Up-Regulated |
| C1orf54 | Up-Regulated |
| LILRB5 | Up-Regulated |
| CD5 | Up-Regulated |
| CLEC12A | Up-Regulated |
| CD3G | Up-Regulated |
| RASGRP4 | Up-Regulated |
| IL7R | Up-Regulated |
| C19orf35 | Up-Regulated |
| CD209 | Up-Regulated |
| TLR4 | Up-Regulated |
| GPR109B | Up-Regulated |
| MS4A7 | Up-Regulated |
| CXCL10 | Up-Regulated |
| CCL21 | Up-Regulated |
| IL16 | Up-Regulated |
| GPR109A | Up-Regulated |
| SLC7A7 | Up-Regulated |
| LCP2 | Up-Regulated |
| IL10RA | Up-Regulated |
| SERPINE1 | Up-Regulated |
| RNASE1 | Up-Regulated |
| TNFRSF13B | Up-Regulated |
| CD96 | Up-Regulated |
| SLC1A3 | Up-Regulated |
| CCL5 | Up-Regulated |
| CCDC69 | Up-Regulated |
| CD1E | Up-Regulated |
| PTAFR | Up-Regulated |
| EIF1AY | Up-Regulated |
| DPEP2 | Up-Regulated |
| KIAA0125 | Up-Regulated |
| RLTPR | Up-Regulated |
| EMR3 | Up-Regulated |
| CDH23 | Up-Regulated |
| GLIPR1 | Up-Regulated |
| HLA-DMA | Up-Regulated |
| DTHD1 | Up-Regulated |
| NLRP1 | Up-Regulated |
| PPP1R16B | Up-Regulated |
| GAS7 | Up-Regulated |
| SIGLEC5 | Up-Regulated |
| C14orf139 | Up-Regulated |
| HLA-DRB1 | Up-Regulated |
| PDCD1 | Up-Regulated |
| ADH1B | Up-Regulated |
| ATP6V0D2 | Up-Regulated |
| TRAF3IP3 | Up-Regulated |
| LPL | Up-Regulated |
| MYO1F | Up-Regulated |
| CD300LF | Up-Regulated |
| CD8B | Up-Regulated |
| HS3ST2 | Up-Regulated |
| CD84 | Up-Regulated |
| FCER2 | Up-Regulated |
| LYZ | Up-Regulated |
| RARRES1 | Up-Regulated |
| MEI1 | Up-Regulated |
| LAPTM5 | Up-Regulated |
| OLFML3 | Up-Regulated |
| LCP1 | Up-Regulated |
| DOK2 | Up-Regulated |
| PRKCH | Up-Regulated |
| HAPLN3 | Up-Regulated |
| PDGFRA | Up-Regulated |
| MPEG1 | Up-Regulated |
| COL6A3 | Up-Regulated |
| CCR5 | Up-Regulated |
| FCGR1A | Up-Regulated |
| FPR2 | Up-Regulated |
| MYO1G | Up-Regulated |
| CRTAC1 | Up-Regulated |
| SELM | Up-Regulated |
| GIMAP5 | Up-Regulated |
| CSF2RA | Up-Regulated |
| PLB1 | Up-Regulated |
| HLA-DQB2 | Up-Regulated |
| CLEC4A | Up-Regulated |
| IRF8 | Up-Regulated |
| ICOS | Up-Regulated |
| AIM2 | Up-Regulated |
| FOSB | Up-Regulated |
| IL1B | Up-Regulated |
| PKD2L1 | Up-Regulated |
| PREX1 | Up-Regulated |
| MARCO | Up-Regulated |
| CXCR6 | Up-Regulated |
| FCRLA | Up-Regulated |
| HLA-DOB | Up-Regulated |
| IGSF6 | Up-Regulated |
| CD200R1 | Up-Regulated |
| HPSE | Up-Regulated |
| PDCD1LG2 | Up-Regulated |
| C1orf127 | Up-Regulated |
| FCER1G | Up-Regulated |
| SIGLEC11 | Up-Regulated |
| NLRP12 | Up-Regulated |
| LY9 | Up-Regulated |
| HSD11B1 | Up-Regulated |
| LOC647121 | Up-Regulated |
| PLAC8 | Up-Regulated |
| C4BPA | Up-Regulated |
| C1QA | Up-Regulated |
| SAA2 | Up-Regulated |
| PTGS1 | Up-Regulated |
| HAMP | Up-Regulated |
| DCN | Up-Regulated |
| CFP | Up-Regulated |
| NOD2 | Up-Regulated |
| MFNG | Up-Regulated |
| GPR68 | Up-Regulated |
| FUT7 | Up-Regulated |
| KIF21B | Up-Regulated |
| PRELP | Up-Regulated |
| KCNJ5 | Up-Regulated |
| TREM1 | Up-Regulated |
| TMEM119 | Up-Regulated |
| SLC13A3 | Up-Regulated |
| CLIC2 | Up-Regulated |
| GBP5 | Up-Regulated |
| SEMA6B | Up-Regulated |
| CD69 | Up-Regulated |
| PDE1B | Up-Regulated |
| GNGT2 | Up-Regulated |
| CTHRC1 | Up-Regulated |
| SIGLEC7 | Up-Regulated |
| PIK3R6 | Up-Regulated |
| ZNF804A | Up-Regulated |
| FAM70A | Up-Regulated |
| BCAT1 | Up-Regulated |
| P2RX7 | Up-Regulated |
| VNN2 | Up-Regulated |
| CLEC10A | Up-Regulated |
| CCL3 | Up-Regulated |
| SERPINA3 | Up-Regulated |
| TM4SF19 | Up-Regulated |
| CSF1R | Up-Regulated |
| RBP1 | Up-Regulated |
| MLPH | Up-Regulated |
| NPL | Up-Regulated |
| CALHM2 | Up-Regulated |
| CDA | Up-Regulated |
| ME1 | Up-Regulated |
| AOAH | Up-Regulated |
| ZFY | Up-Regulated |
| KCNN4 | Up-Regulated |
| HAVCR2 | Up-Regulated |
| PLAUR | Up-Regulated |
| TEX11 | Up-Regulated |
| TNFAIP8L2 | Up-Regulated |
| APOL3 | Up-Regulated |
| IL2RB | Up-Regulated |
| CD3E | Up-Regulated |
| FAM180A | Up-Regulated |
| CP | Up-Regulated |
| LY96 | Up-Regulated |
| SFRP1 | Up-Regulated |
| DAPL1 | Up-Regulated |
| SCML4 | Up-Regulated |
| CD40LG | Up-Regulated |
| CES1 | Up-Regulated |
| HGF | Up-Regulated |
| GGT5 | Up-Regulated |
| CFD | Up-Regulated |
| APOC1 | Up-Regulated |
| C1orf162 | Up-Regulated |
| ITGAL | Up-Regulated |
| THEMIS | Up-Regulated |
| VAV1 | Up-Regulated |
| HTRA4 | Up-Regulated |
| ANKRD58 | Up-Regulated |
| CSF2RB | Up-Regulated |
| HLX | Up-Regulated |
| KLRB1 | Up-Regulated |
| DNASE2B | Up-Regulated |
| ASB2 | Up-Regulated |
| CD1C | Up-Regulated |
| C19orf59 | Up-Regulated |
| HCLS1 | Up-Regulated |
| SLFN12L | Up-Regulated |
| S100A9 | Up-Regulated |
| HOPX | Up-Regulated |
| MMP9 | Up-Regulated |
| IL2RG | Up-Regulated |
| CCL13 | Up-Regulated |
| DERL3 | Up-Regulated |
| ELFN1 | Down-Regulated |
| CHD5 | Down-Regulated |
| CWH43 | Down-Regulated |
| C1QL4 | Down-Regulated |
