# Supplementary material for: Identification of 4-genes model in papillary renal cell tumor microenvironment based on comprehensive analysis
Source: BMC Cancer. 2021 May 17;21:553. doi: 10.1186/s12885-021-08319-0 (PMC8127234; doi:10.1186/s12885-021-08319-0)
Supplement: Supplementary file 3 — Additional file 3: Supplementary Table S2 120 genes associated with PRCC overall survival. [file 12885_2021_8319_MOESM3_ESM.docx]

Title: Identification of 4-genes model in papillary renal cell tumor microenvironment based on comprehensive analysis

Liang Luo^1^*, Haiyi Zhou^2^, Hao Su^1^

1 Department of Urology, The Third Affiliated Hospital, Sun Yat-sen University, Guangzhou, 510630, China

2 Department of Gynecology of traditional Chinese Medicine, Shanxi Academy of Traditional Chinese Medicine, Taiyuan 030000, China

Corresponding author:

Liang Luo, Department of Urology, The Third Affiliated Hospital, Sun Yat-sen University, Tianhe Road 600, Guangzhou, 510630, China

Telephone: +86-20-85252990; Fax: +86-20-85252678

E-mail: luoliang6@mail2.sysu.edu.cn

**Supplementary Table S2**

**120 genes associated with PRCC overall survival**

| Gene | P value |
| --- | --- |
| COL6A3 | 8.93E-07 |
| ADAM19 | 3.60E-06 |
| CXCL13 | 3.83E-06 |
| CTHRC1 | 8.65E-06 |
| FAP | 9.10E-06 |
| IL15RA | 2.07E-05 |
| HAPLN3 | 2.27E-05 |
| PRELP | 2.43E-05 |
| COL22A1 | 2.62E-05 |
| CCL19 | 2.83E-05 |
| SEMA7A | 6.68E-05 |
| LEF1 | 7.09E-05 |
| MFAP5 | 2.81E-04 |
| CCDC80 | 6.52E-04 |
| NRP2 | 7.89E-04 |
| EFEMP1 | 9.83E-04 |
| PODN | 9.92E-04 |
| SEMA6B | 0.001035 |
| FCRL3 | 0.001066 |
| PDCD1 | 0.001285 |
| POSTN | 0.001294 |
| GZMB | 0.001315 |
| TMEM119 | 0.001383 |
| CCL21 | 0.001469 |
| KYNU | 0.001536 |
| FCRL2 | 0.00159 |
| MGP | 0.001621 |
| ISLR | 0.001703 |
| TNFRSF9 | 0.00181 |
| EMILIN1 | 0.002027 |
| CREB3L1 | 0.002271 |
| IL21R | 0.002337 |
| LAG3 | 0.002356 |
| AIM2 | 0.002471 |
| PTGIR | 0.002493 |
| GPR68 | 0.002542 |
| HSD11B1 | 0.002572 |
| RLTPR | 0.002582 |
| S100B | 0.002911 |
| PRR5L | 0.003203 |
| ART4 | 0.004189 |
| CLIC2 | 0.004332 |
| SP140 | 0.004388 |
| TMEM173 | 0.004418 |
| FCRL5 | 0.004478 |
| CD38 | 0.004517 |
| HEPH | 0.005018 |
| SPIB | 0.005078 |
| IL6 | 0.005111 |
| POU2AF1 | 0.00603 |
| CPXM1 | 0.006118 |
| CXCL10 | 0.006387 |
| LOC96610 | 0.007198 |
| CD80 | 0.007531 |
| BST2 | 0.007601 |
| INMT | 0.007734 |
| GPR174 | 0.008209 |
| PRDM8 | 0.008387 |
| HOPX | 0.00868 |
| SELL | 0.008859 |
| GBP4 | 0.009402 |
| HLX | 0.009617 |
| DERL3 | 0.009752 |
| C1S | 0.010848 |
| FAM101A | 0.01127 |
| GRAMD1B | 0.012173 |
| TIGIT | 0.012399 |
| LUM | 0.012499 |
| MS4A1 | 0.012598 |
| CXCR5 | 0.01348 |
| ABCD2 | 0.01352 |
| STEAP4 | 0.013582 |
| PDGFRA | 0.013971 |
| DCN | 0.014072 |
| UBD | 0.014159 |
| CD79B | 0.017009 |
| PNOC | 0.017272 |
| NELL2 | 0.017454 |
| SFRP1 | 0.017759 |
| CAMK4 | 0.018024 |
| CPNE5 | 0.018253 |
| ITGA4 | 0.018741 |
| BLK | 0.019159 |
| CD19 | 0.019791 |
| HTR2B | 0.019938 |
| CD79A | 0.020565 |
| C1R | 0.020774 |
| NIPAL4 | 0.020811 |
| NKG7 | 0.021405 |
| KIAA0125 | 0.022419 |
| AQP9 | 0.022459 |
| SERPINE1 | 0.024061 |
| MGC29506 | 0.02423 |
| UCP2 | 0.024848 |
| MX2 | 0.025384 |
| SH2D2A | 0.025543 |
| ADAM6 | 0.025955 |
| FCRLA | 0.027473 |
| RFTN1 | 0.027652 |
| CALHM2 | 0.028312 |
| AG2 | 0.028648 |
| FMO3 | 0.028648 |
| GGT5 | 0.028945 |
| ITM2A | 0.03256 |
| GPAT2 | 0.03364 |
| VNN2 | 0.033954 |
| SERPINA3 | 0.036211 |
| CXCL11 | 0.036669 |
| WNT10A | 0.037936 |
| OAS2 | 0.038815 |
| ITIH3 | 0.039476 |
| HS3ST3A1 | 0.039928 |
| CP | 0.042131 |
| TSPAN8 | 0.043871 |
| LOC647121 | 0.044087 |
| CD8A | 0.046976 |
| CD44 | 0.048274 |
| KCNN4 | 0.048292 |
| ST8SIA4 | 0.049352 |
| F13A1 | 0.049867 |
