# Supplementary material for: Identification of 4-genes model in papillary renal cell tumor microenvironment based on comprehensive analysis
Source: BMC Cancer. 2021 May 17;21:553. doi: 10.1186/s12885-021-08319-0 (PMC8127234; doi:10.1186/s12885-021-08319-0)
Supplement: Supplementary file 5 — Additional file 5: Supplementary Table S3 Univariate cox proportional hazard regression analysis of 22 genes based on TCGA database. [file 12885_2021_8319_MOESM5_ESM.docx]

Title: Identification of 4-genes model in papillary renal cell tumor microenvironment based on comprehensive analysis

Liang Luo^1^*, Haiyi Zhou^2^, Hao Su^1^

1 Department of Urology, The Third Affiliated Hospital, Sun Yat-sen University, Guangzhou, 510630, China

2 Department of Gynecology of traditional Chinese Medicine, Shanxi Academy of Traditional Chinese Medicine, Taiyuan 030000, China

Corresponding author:

Liang Luo, Department of Urology, The Third Affiliated Hospital, Sun Yat-sen University, Tianhe Road 600, Guangzhou, 510630, China

Telephone: +86-20-85252990; Fax: +86-20-85252678

E-mail: luoliang6@mail2.sysu.edu.cn

**Supplementary Table S3**

**Univariate cox proportional hazard regression analysis of 22 genes based on TCGA database**

| Gene | HR | Z | P value |
| --- | --- | --- | --- |
| CXCR5 | 1.233632 | 2.471236 | 0.013465 |
| CD19 | 1.151194 | 2.330043 | 0.019804 |
| PDCD1 | 1.279521 | 3.219614 | 0.001284 |
| IL21R | 1.300843 | 3.045696 | 0.002321 |
| GZMB | 1.29393 | 3.218895 | 0.001287 |
| TNFRSF9 | 1.254914 | 3.121262 | 0.001801 |
| CD38 | 1.271727 | 2.841679 | 0.004488 |
| CXCL10 | 1.233098 | 2.726562 | 0.0064 |
| SELL | 1.319797 | 2.61736 | 0.008861 |
| LAG3 | 1.234082 | 3.041179 | 0.002357 |
| CD44 | 1.210629 | 1.97582 | 0.048175 |
| CD80 | 1.358777 | 2.670573 | 0.007572 |
| CCL21 | 1.15497 | 3.185821 | 0.001443 |
| ITGA4 | 1.333624 | 2.352581 | 0.018644 |
| CCL19 | 1.206037 | 4.191764 | 2.77E-05 |
| IL6 | 1.171632 | 2.808505 | 0.004977 |
| CXCL13 | 1.238469 | 4.622631 | 3.79E-06 |
