# Supplementary material for: Identification of 4-genes model in papillary renal cell tumor microenvironment based on comprehensive analysis
Source: BMC Cancer. 2021 May 17;21:553. doi: 10.1186/s12885-021-08319-0 (PMC8127234; doi:10.1186/s12885-021-08319-0)
Supplement: Supplementary file 6 — Additional file 6: Supplementary Table S4 Multivariate cox regression results of 4-gene prognostic model. [file 12885_2021_8319_MOESM6_ESM.docx]

Title: Identification of 4-genes model in papillary renal cell tumor microenvironment based on comprehensive analysis

Liang Luo^1^*, Haiyi Zhou^2^, Hao Su^1^

1 Department of Urology, The Third Affiliated Hospital, Sun Yat-sen University, Guangzhou, 510630, China

2 Department of Gynecology of traditional Chinese Medicine, Shanxi Academy of Traditional Chinese Medicine, Taiyuan 030000, China

Corresponding author:

Liang Luo, Department of Urology, The Third Affiliated Hospital, Sun Yat-sen University, Tianhe Road 600, Guangzhou, 510630, China

Telephone: +86-20-85252990; Fax: +86-20-85252678

E-mail: luoliang6@mail2.sysu.edu.cn

**Supplementary Table S4**

**Multivariate cox regression results of 4-gene prognostic model**

| Gene symbol | Coef | Exp(coef) | Se(coef) | Z | P value |
| --- | --- | --- | --- | --- | --- |
| CD79A | -0.2402 | 0.78647 | 0.10393 | -2.311 | 0.020823 |
| CXCL13 | 0.22031 | 1.24646 | 0.06644 | 3.316 | 0.000914 |
| IL6 | 0.12025 | 1.12778 | 0.06464 | 1.86 | 0.062824 |
| CCL19 | 0.17997 | 1.19718 | 0.07393 | 2.435 | 0.014912 |
